# Metabolic and Genetic Markers of Biological Age

**DOI:** 10.3389/fgene.2017.00064

**Published:** 2017-05-23

**Authors:** S. Michal Jazwinski, Sangkyu Kim

**Affiliations:** Tulane Center for Aging, Department of Medicine, Tulane University Health Sciences Center, Tulane School of Medicine, New OrleansLA, United States

**Keywords:** healthy aging, frailty index, energy metabolism, genes, DNA methylation

## Abstract

Biological age is a concept that takes into account the heterogeneity of the aging process in different individuals that results in differences in survival and variations in relative health. Any measure of biological age must be better than chronological age at predicting mortality. Several quantitative measures of biological age have been developed. Among them are frailty indices, one of which called FI_34_ is discussed here in greater detail. FI_34_ increases exponentially with age reflecting decline in health and function ability. It readily depicts different patterns and trajectories of aging, and it is moderately heritable. Thus, it has been used to identify a genomic region on chromosome 12 associated with healthy aging. FI_34_ has also been useful in describing the metabolic characteristics of this phenotype, revealing both sex and genetic differences. These differences give rise to specific, testable models regarding healthy aging, which involve cell and tissue damage and mitochondrial metabolism. FI_34_ has been directly compared to various metrics based on DNA methylation as a predictor of mortality, demonstrating that it outperforms them uniformly. This and other frailty indices take a top-down, systems based view of aging that is cognizant of the integrated function of the complex aging system.

## Introduction

We all notice the passage of time when we inspect our faces in the mirror as the years go by. For many, this progression is punctuated by disease, and often advancing age is marked by chronic disease and degeneration. Indeed, the major risk factor for diseases such as macular degeneration, type 2 diabetes, atherosclerosis, cancer, pulmonary disease, Alzheimer’s disease, osteoporosis, and arthritis is aging (Sierra, 2016). All of these diseases are accompanied by chronic inflammation, but we do not know whether this inflammation is a cause or a consequence of these maladies. What has become clear over the past decade or so is that the contributors to these disorders at the cellular level are damaged molecules and organelles (Lopez-Otin et al., 2013).

Aging at the cellular level translates to aging of the organism at the physiologic level. Physiologic functions decline with age. Nerve conductance velocity, maximum heart rate, kidney function, pulmonary function, and maximum aerobic capacity all decline, at a greater rate with age in more or less that order (Shock, 1967). Cognitive function in the absence of disease does not escape this rule. However, crystallized intelligence (vocabulary) tends to remain constant throughout adulthood, while fluid intelligence which encompasses processing speed, working memory, and long-term memory appears to decrease monotonically starting at age 20 (Park and Bischof, 2013). This cross-sectional view of cognition during the lifespan masks marked individual variation in cognitive decline (McArdle, 2011). Individuals show trajectories that include both increases and decreases in cognitive function even in very old age. These individual differences suggest that genetic and environmental factors affect functional decline with age. For example, decline in learning ability during aging is modulated by the *𝜀4* allele of *APOE* (Papenberg et al., 2015). This allele also interacts with life events, an environmental factor, to impact negative affect in centenarians (Martin et al., 2014). Individual profiles in age-related changes are also found for physical function, just as they are for cognitive function.

The organism is complex presenting itself as a non-linear dynamic system. This is because it is composed of a multitude of interacting components. During aging, this complexity declines; in other words, the number of interactions declines. At the level of various physiologic systems in the body, this translates into a transition from a highly irregular output to a less complex and predictable pattern. In terms of function ability, this is mirrored in a decrease in functionality, and with time it results in frailty. Thus, the interactions between body components lead to emergent properties at higher levels of organization that are not simply the sum of the component parts (Sleimen-Malkoun et al., 2014). At all levels, there is a decline in connectivity and functionality with age, rendering the organism less robust and resilient.

There are tipping points in a human’s lifespan at which the individual can continue to age healthily/successfully or decline into frailty and finally disability. A systems approach is necessary to understand this transition. This conceptualization is not just a theoretical construct. It has been shown that there are at least five distinct trajectories of disability during the last year in the life of nonagenarians (Gill et al., 2010). These include no disability, catastrophic disability, accelerated disability, progressive disability, and persistently severe disability. It is important to identify the genetic and environmental factors that determine which trajectory an individual will take. In order to do this, we need a top-down measure that quantitatively describes the biological age of an organism. Several approaches for quantification of biological age have been proposed, and they are mentioned below. This provides context for the remainder of this article which describes a measure of healthy aging, FI_34_, which we have used extensively for the genetic and phenotypic characterization of this phenotype, with a focus on energy metabolism.

## Quantitative Measures of Biological Age

Several quantitative measures of biological age have been proposed and used to varying degrees in the past. Many of them discriminate healthy from unhealthy aging. Centenarians have been categorized as survivors, delayers, or escapers, on the basis of the presence of disease (Evert et al., 2003). However, this classification is not generally useful because most individuals do suffer from various disorders as they age. Successful aging has been quantified as a low level of disease and disability, relatively high physical and cognitive functioning, and an active engagement in life activities (Rowe and Kahn, 1997). On the other hand, the clinical syndrome of frailty is defined by the presence of at least three of the five deficits: weight loss, exhaustion, muscle weakness, slow gait, and low physical activity (Fried et al., 2001). This frailty phenotype assumes a compromise in functional reserve and dysregulation that predisposes to the inability to recover from stress and can lead to disability.

Frailty indices, also called deficit indices, have been widely used in aging studies. They incorporate as many as 100 health and function variables that access a broad array of physiologic systems (Mitnitski et al., 2001; Rockwood and Mitnitski, 2007; Rockwood et al., 2007). They sum these deficits and grade the individual on a scale of 0–1, in which the latter denotes the presence of all deficits. The utility of this approach is that it takes into account the heterogeneity of aging across individuals. In addition to this uncomplicated treatment, multivariate frailty indices have been applied using a clustering approach to define different forms of frailty (Dato et al., 2012).

Biomarkers have been used in the place of deficits to fashion measures of biological aging. Allostatic load conceptualizes the cumulative biological burden as the body adapts to life stress. It is operationalized through the measurement of 10 or more biomarkers that are meant to assess several regulatory systems and processes (Seeman et al., 2001). Biomarkers have also been used in a statistical approach that fits them optimally to age, across all biomarkers; this procedure works well when chronological age is used to center this fitting (Levine, 2013). A similar approach has been applied to longitudinal data to generate estimates of both biological age and the rate of biological aging (Belsky et al., 2015). A different application of biomarkers involves their use in a multivariate measure of departure from the centroid of a healthy-aging population (Milot et al., 2014). Hierarchical clustering approaches have also been applied to identify patterns of biomarker age changes that predict survival (Sebastiani et al., 2017). Recently, DNA methylation markers have been used in the place of serum biomarkers to generate multivariate measures of age (Hannum et al., 2013; Horvath, 2013).

Biomarkers are actually used as endophenotypes of the organismal aging phenotype. Their use assumes that they reflect a biological process that results in the manifestations of aging of the organism that increase the risk of mortality. Serum biomarkers are not the only kind of biomarkers that have been applied to biological aging. Suites composed of measures of physical and cognitive function, as well as physical examination and laboratory values have been used to identify principal components that are endophenotypes of a long and healthy life (Matteini et al., 2010).

## Derivation and Properties of FI_34_

A frailty index composed of 34 health and physical and cognitive function variables, called FI_34_ has been developed (Kim et al., 2013). It includes 34 symptoms, diseases, and disabilities, and it is expressed as a fraction, from 0 to 1, of the total presented by an individual (Supplementary Table 1). Its validation and use in the study of healthy aging has been documented, and this is briefly reviewed below because of the value of FI_34_ in the development of an understanding of human healthy aging at the genetic and phenotypic levels. FI_34_ is better than chronological age as a predictor of mortality. Indeed, there is a 27% increase in the hazard of death for every 0.1 increase in FI_34_ (*p* < 0.004), while for every 1.0 year of age this hazard increases 1.7% (*p* = 0.124), by Cox regression (uncensored). This is important because any measure of biological age must be better than chronological age at predicting survival to be of value in quantitating the heterogeneity in aging of individuals in the population. FI_34_ is a measure of healthy aging, as lower values of this metric represent fewer health and function deficits.

FI_34_ increases with chronological age across a population, and this increase is best described by an exponential function (see Kim et al., 2013). The increase in FI_34_ with age was compared for the offspring of long- and short-lived parents. The former had at least one parent who lived past the age of 90, while in the latter neither parent lived past the age of 75. Interestingly, the rate of increase in FI_34_ was greater for the offspring of short-lived parents at any given age. For example, it was 20% compared to 12% for offspring of short- and long-lived parents, respectively, at age 70. Overall, FI_34_ was 63% (*p* = 0.0002) larger in the former, across all ages. Hierarchical clustering of the variables in FI_34_ reveals that the patterns of aging they describe are visibly different for offspring of short- and long-lived parents. Not surprisingly then, FI_34_ is moderately heritable, with an additive (narrow sense) genetic component of 0.39. Consistent with the heterogeneity of individual age-trajectories of physical and cognitive function, mentioned earlier, longitudinal analysis of FI_34_ across individuals displays substantial plasticity (Kim and Jazwinski, 2015). This plasticity suggests the operation of both genetic and environmental factors, raising the possibility of manipulating healthy aging.

## Genetic Contributions to Healthy Aging

Based on its heritability, FI_34_ has been used in a genome-wide linkage scan to search for loci associated with healthy aging (Kim et al., 2015). One such quantitative trait locus (QTL) was found on chromosome 12 (12q13-14). Fine mapping of this locus was then performed in a different population, using over 300 single-nucleotide polymorphisms (SNP). This divided the 1 Mb QTL into three healthy aging-associated sites (HAS). As it turned out, many of these SNP are also associated with longevity in a highly significant manner (Kim et al., 2015).

HAS-1 (∼20 kb), HAS-2 (∼140 kb), and HAS-3 (∼70 kb) are located in intergenic regions at 12q13-14 (Kim et al., 2015). HAS-1 has previously been associated with several diseases (coeliac, type 1 diabetes, rheumatoid arthritis, multiple sclerosis), and it has protein-coding genes in its vicinity. It has the features of an enhancer, and this function has been validated experimentally in multiple cell lines. Among the regulatory signatures in this region are histone modifications characteristic of regulatory elements including promoters, transcription factor binding sites, DNase I hypersensitive sites, and CpG islands. One of the signature SNP is located in a CCAAT/enhancer binding protein (C/EBP) binding site, displaying enhancer activity in luciferase reporter assays. HAS-2 also possesses marks typical of a strong enhancer, but it lacks the histone modifications typical of promoters which is consistent with the lack of protein-coding genes in its vicinity. The chromatin features of HAS-2 coincide with enhancer activity across several cell lines. Several SNP in HAS-2 are associated with transcription of genes on different chromosomes. On the other hand, HAS-3 has the chromatin features of a repressor. It has a long intergenic non-coding RNA (lincRNA) sequence, and it contains multiple Polycomb-repressed blocks in one cell line. Two genes flanking this genomic region are validated targets of Polycomb silencing. It would appear that HAS-2 and HAS-3 may act on distant genomic regions, as enhancer and repressor, respectively. Altogether, the genomic annotations recited here conjure up the operation of regulatory elements that affect the expression of a network of genes associated with healthy aging.

## Metabolic Phenotype Associated With FI_34_

Ultimately, our goal is to understand the factors that contribute to a healthy aging trajectory, as opposed to one that leads to disability. We wondered whether there is an energetic cost associated with healthy aging. Total energy expenditure (TEE) is composed of three components: resting metabolic rate (RMR), activity energy expenditure (AEE), and diet-induced thermogenesis. These components make up 60–70%, 20–30%, and about 10% of TEE, respectively. AEE and maximum aerobic capacity (VO_2_ max) together make up the energy reserve, which is the total energy that can be devoted to physical activity. TEE, RMR, and AEE all decline with age (Kim and Jazwinski, 2015).

FI_34_ increases with age, so one would expect that TEE, RMR, and AEE measures of energy metabolism would have a reciprocal relationship to FI_34_. However, RMR increases as FI_34_ becomes larger (Kim et al., 2014). This association is maintained even after adjustment for age, sex, body composition, circulating insulin-like growth factor 1 (IGF-1), circulating thyroid hormones (T3 and T4), and TEE. It still remains when circulating creatine kinase (CK) levels are included as a covariate. In fact, body composition in the form of fat-free mass (FFM) and fat mass (FM), as well as CK, are significant predictors of FI_34_ along with RMR.

**FIGURE 1:**
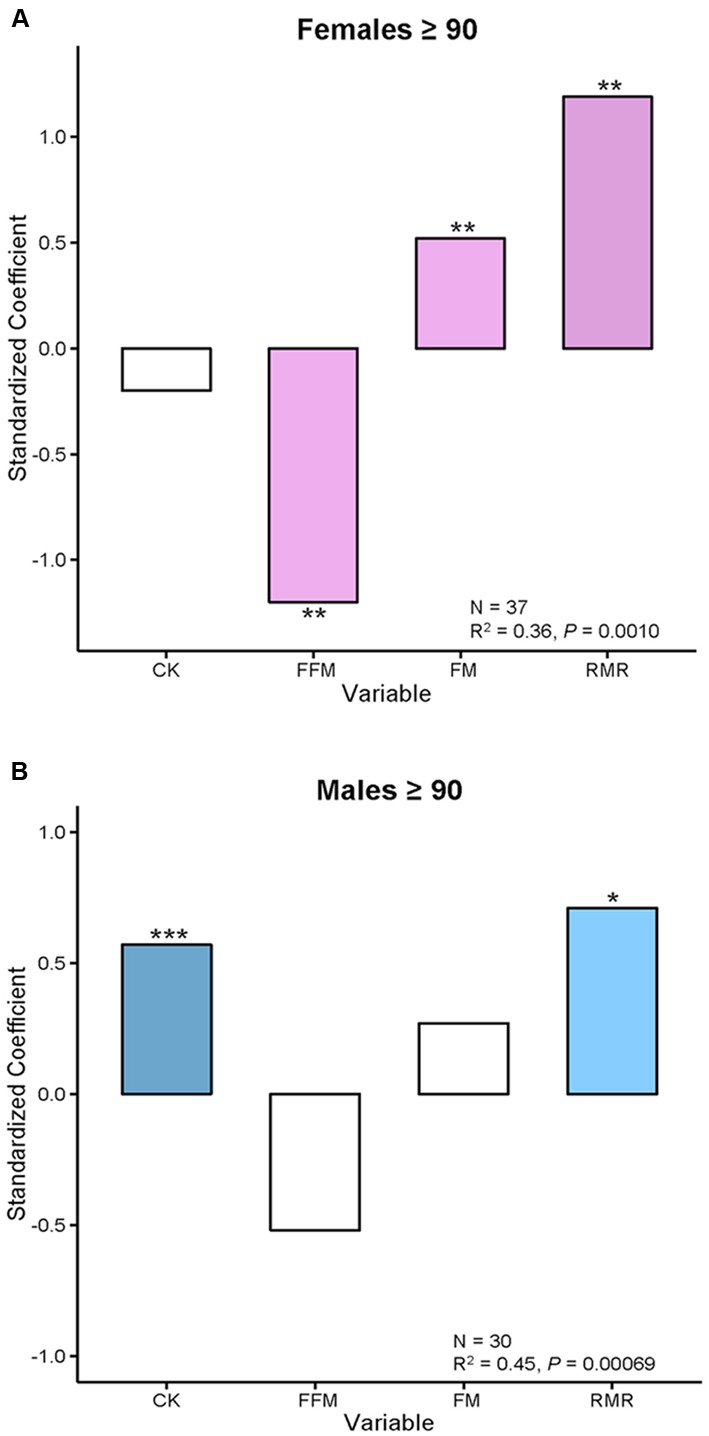
**Body composition and creatine kinase levels are associated with FI_34_ in females and males, respectively. (A)** Female and **(B)** male nonagenarians were compared. FI_34_ is the outcome variable presented as a standardized coefficient in multiple linear regressions. The explanatory variables are CK, FFM, FM, and RMR. The sample size (*N*) and the coefficient of determination (*R*^2^) with *p*-value are shown. ^∗^*p* < 0.05; ^∗∗^*p* < 0.01; ^∗∗∗^*p* < 0.001, for the standardized coefficients for each variable. Based on data from Kim et al. (2014).

The complicated association of FI_34_ and RMR gains some clarity when it is separately examined in males and females (**Figure 1**). It becomes evident that in the nonagenarians examined here the positive relationship of FI_34_ and RMR plays out somewhat differently in males and females. In the former, CK levels are also involved, while in females it is body composition (FFM and FM). Clearly, the overall age-related decline in RMR is only applicable to healthy nonagenarians, while in unhealthy ones (higher FI_34_) it is reversed. It has been proposed that this RMR increase constitutes a device to maintain homeodynamics in the oldest-old as their health deteriorates (Kim and Jazwinski, 2015).

In sum, there is a metabolic cost to unhealthy aging/frailty in both males and females. It manifests itself as an increase in RMR for maintenance of integrated body function. This constitutes a metabolic compensation that allows the organism to make do in a worsening situation. The increase in RMR in males is associated with an increase in circulating CK levels, which are a measure of tissue damage. In females, increased RMR is associated with a decline in muscle mass (FFM). Furthermore, females display a related decrease in physical activity levels (Kim and Jazwinski, 2015).

## Genetic Inputs to the Metabolic Phenotype of FI_34_

There are also genetic differences underlying the differences in association of FI_34_ and RMR in males and females. Circulating CK levels in males (not females) are associated directly with cardiac problems and inversely with healthy kidney function, supporting the notion that CK levels reflect tissue damage (Kim et al., 2016b). Physical activity appears to have a protective effect. Potential regulatory variants in the genes *XRCC6* and *LASS1* are associated with lower circulating CK levels in males but not in females (Kim et al., 2016b). [*XRCC6* has been associated with telomere length during aging (Kim et al., 2012), and *LASS1* has been associated with longevity and healthy aging (Jazwinski et al., 2010).] Interestingly, *XRCC6* encodes Ku70 which is known to prevent cell death by binding Bax, a protein that activates a mitochondrial cell death pathway. *LASS1* encodes ceramide synthase whose product ceramide triggers apoptosis in caspase-dependent and -independent pathways. This prompts a model in which metabolic and genomic stress associated with unhealthy aging in males lead to a decrease in Xrcc6p and increase in Lass1p activity resulting in cell death, and this is associated with increased heart problems, decreased kidney function, and other damage, and the related increase in RMR that we found. The effects of the variants in the two genes on transcription are consistent with this model.

In females, the genetic factors contributing to the observed increased RMR when FI_34_ increases are different than in males (Kim et al., 2016a). Mitochondrial activity appears to be involved more directly. Regulatory variants in the promoter of the *UCP2* gene that can affect transcription factor binding and expression of the gene are associated with FI_34_. This association is not found in males. The mitochondrial uncoupling protein Ucp2 encodes a mitochondrial inner membrane transporter (Vozza et al., 2014). Increased Ucp2 activity portends a switch from glucose to glutamine utilization as fuel, and it coincidentally lowers mitochondrial membrane potential and the ATP:ADP ratio. Variation in the uncoupling protein gene *UCP3* is also associated with FI_34_ in females but not in males. The SNP examined is located in the 3′-untranslated region (3′-UTR) of the gene, and it is known to increase its expression. This SNP interacts with RMR to affect FI_34_, suggesting that the net effect is to increase the rate or intensity of mitochondrial activity (Kim et al., 2016a). These alterations in mitochondrial metabolism promoted by increased Ucp2 and Ucp3 protein activities would compensate for loss of muscle mass in females. The genetic analyses described here further support the importance of energy metabolism in healthy aging.

## FI_34_ and Dna Methylation Age as Measures of Biological Age

**FIGURE 2:**
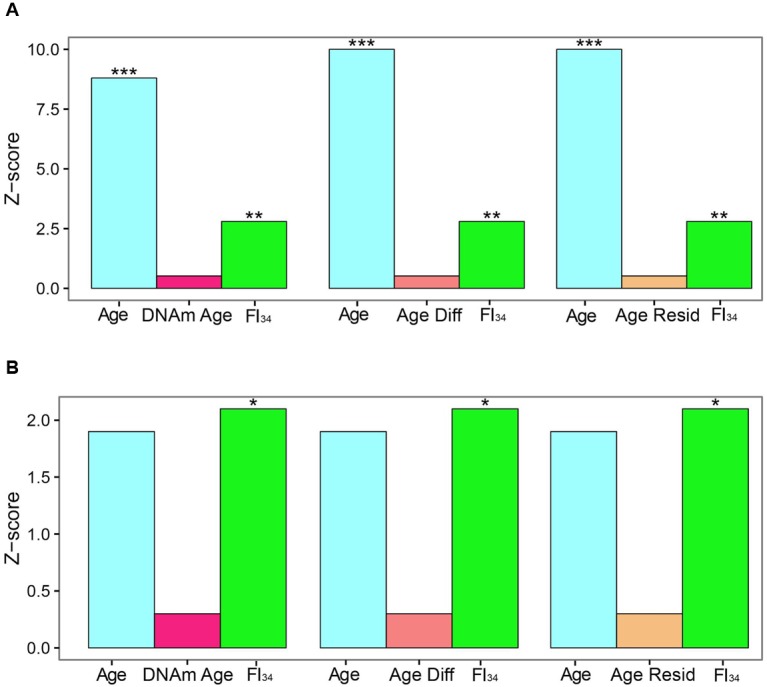
**Comparison of effect sizes for hazards of death.** Cox proportional hazards regressions were applied to the survival data of 262 subjects ages 60–103 and are presented as *Z* scores. Age, FI_34_, DNA methylation age (DNAm Age), Age Acceleration Difference (Age Diff), and Age Acceleration Residual (Age Resid) are the covariates. **(A)** All subjects, **(B)** nonagenarians (*N* = 161). ^∗^*p* < 0.05; ^∗∗^*p* < 0.01; ^∗∗∗^*p* < 0.001. This figure has been adapted from Kim et al. (2017) under the Creative Commons Attribution 4.0 International License (http://creativecommons.org/licenses/by/4.0/).

DNA methylation markers have been proposed as measures of aging. In particular, DNA Methylation Age and its derivatives Age Acceleration Difference and Age Acceleration Residual, based on methylation at 353 specific CpG sites have gained particular traction recently (Horvath, 2013; Horvath et al., 2015; Marioni et al., 2015; Chen et al., 2016; Christiansen et al., 2016; Perna et al., 2016). They have been compared side-by-side recently as measures of biological age, based on their ability to predict survival (Kim et al., 2017). In the presence of age as a covariate, these DNA methylation related measures were not significant predictors of survival, while FI_34_ retained significance. In fact, it retained significance even in the presence of these DNA methylation related measures (**Figure 2**). What is more, only FI_34_ was a predictor of survival in nonagenarians; age itself was not a predictor. Thus, FI_34_ is a robust predictor of the hazard of death, containing information that age alone does not, while the DNA methylation related measures merely reflect chronological age with no significant added information. This suggests that a firm biological rationale is important before sophisticated statistical treatment is applied.

## Author Contributions

SMJ organized the article and generated the first draft with materials provided by SK, who reviewed this draft and edited figures.

## Conflict of Interest Statement

The authors declare that the research was conducted in the absence of any commercial or financial relationships that could be construed as a potential conflict of interest.

## References

[B1] Belsky D. W. Caspi A. Houts R. Cohen H. J. Corcoran D. L. Danese A. (2015). Quantification of biological aging in young adults. *Proc. Natl. Acad. Sci. U.S.A.* 112 E4104–E4110. 10.1073/pnas.1506264112 26150497 PMC4522793

[B2] Chen B. H. Marioni R. E. Colicino E. Peters M. J. Ward-Caviness C. K. Tsai P. C. (2016). DNA methylation-based measures of biological age: meta-analysis predicting time to death. *Aging (Albany NY)* 8 1844–1865. 10.18632/aging.101020 27690265 PMC5076441

[B3] Christiansen L. Lenart A. Tan Q. Vaupel J. W. Aviv A. McGue M. (2016). DNA methylation age is associated with mortality in a longitudinal Danish twin study. *Aging Cell* 15 149–154. 10.1111/acel.12421 26594032 PMC4717264

[B4] Dato S. Montesanto A. Lagani V. Jeune B. Christensen K. Passarino G. (2012). Frailty phenotypes in the elderly based on cluster analysis: a longitudinal study of two Danish cohorts. Evidence for a genetic influence on frailty. *Age (Dordr)* 34 571–582. 10.1007/s11357-011-9257-x 21567248 PMC3337941

[B5] Evert J. Lawler E. Bogan H. Perls T. (2003). Morbidity profiles of centenarians: survivors, delayers, and escapers. *J. Gerontol. A Biol. Sci. Med. Sci.* 58 232–237. 12634289 10.1093/gerona/58.3.m232

[B6] Fried L. P. Tangen C. M. Walston J. Newman A. B. Hirsch C. Gottdiener J. (2001). Frailty in older adults: evidence for a phenotype. *J. Gerontol. A Biol. Sci. Med. Sci.* 56 M146–M156. 11253156 10.1093/gerona/56.3.m146

[B7] Gill T. M. Gahbauer E. A. Han L. Allore H. G. (2010). Trajectories of disability in the last year of life. *N. Engl. J. Med.* 362 1173–1180. 10.1056/NEJMoa0909087 20357280 PMC2877372

[B8] Hannum G. Guinney J. Zhao L. Zhang L. Hughes G. Sadda S. (2013). Genome-wide methylation profiles reveal quantitative views of human aging rates. *Mol. Cell.* 49 359–367. 10.1016/j.molcel.2012.10.016 23177740 PMC3780611

[B9] Horvath S. (2013). DNA methylation age of human tissues and cell types. *Genome Biol.* 14:R115 10.1186/gb-2013-14-10-r115 PMC4015143 24138928

[B10] Horvath S. Pirazzini C. Bacalini M. G. Gentilini D. Di Blasio A. M. Delledonne M. (2015). Decreased epigenetic age of PBMCs from Italian semi-supercentenarians and their offspring. *Aging (Albany NY)* 7 1159–1170. 10.18632/aging.100861 26678252 PMC4712339

[B11] Jazwinski S. M. Kim S. Dai J. Li L. Bi X. Jiang J. C. (2010). HRAS1 and LASS1 with APOE are associated with human longevity and healthy aging. *Aging Cell* 9 698–708. 10.1111/j.1474-9726.2010.00600.x 20569235 PMC2941558

[B12] Kim S. Bi X. Czarny-Ratajczak M. Dai J. Welsh D. A. Myers L. (2012). Telomere maintenance genes SIRT1 and XRCC6 impact age-related decline in telomere length but only SIRT1 is associated with human longevity. *Biogerontology* 13 119–131. 10.1007/s10522-011-9360-5 21972126 PMC3272146

[B13] Kim S. Jazwinski S. M. (2015). Quantitative measures of healthy aging and biological age. *Healthy Aging Res.* 4:26 10.12715/har.2015.4.26 PMC4440677 26005669

[B14] Kim S. Myers L. Ravussin E. Cherry K. E. Jazwinski S. M. (2016a). Single nucleotide polymorphisms linked to mitochondrial uncoupling protein genes UCP2 and UCP3 affect mitochondrial metabolism and healthy aging in female nonagenarians. *Biogerontology* 17 725–736. 10.1007/s10522-016-9643-y 26965008 PMC4935613

[B15] Kim S. Myers L. Wyckoff J. Cherry K. E. Jazwinski S. M. (2017). The frailty index outperforms DNA methylation age and its derivatives as an indicator of biological age. *GeroScience* 39 83–92. 10.1007/s11357-017-9960-3 28299637 PMC5352589

[B16] Kim S. Simon E. Myers L. Hamm L. L. Jazwinski S. M. (2016b). Programmed cell death genes are linked to elevated creatine kinase levels in unhealthy male nonagenarians. *Gerontology* 62 519–529. 10.1159/000443793 26913518 PMC4993668

[B17] Kim S. Welsh D. A. Cherry K. E. Myers L. Jazwinski S. M. (2013). Association of healthy aging with parental longevity. *Age (Dordr)* 35 1975–1982. 10.1007/s11357-012-9472-0 22986583 PMC3776103

[B18] Kim S. Welsh D. A. Myers L. Cherry K. E. Wyckoff J. Jazwinskik S. M. (2015). Non-coding genomic regions possessing enhancer and silencer potential are associated with healthy aging and exceptional survival. *Oncotarget* 6 3600–3612. 10.18632/oncotarget.2877 25682868 PMC4414140

[B19] Kim S. Welsh D. A. Ravussin E. Welsch M. A. Cherry K. E. Myers L. (2014). An elevation of resting metabolic rate with declining health in nonagenarians may be associated with decreased muscle mass and function in women and men, respectively. *J. Gerontol. A Biol. Sci. Med. Sci.* 69 650–656. 10.1093/gerona/glt150 24162336 PMC4022095

[B20] Levine M. E. (2013). Modeling the rate of senescence: can estimated biological age predict mortality more accurately than chronological age? *J. Gerontol. A Biol. Sci. Med. Sci.* 68 667–674. 10.1093/gerona/gls233 23213031 PMC3660119

[B21] Lopez-Otin C. Blasco M. A. Partridge L. Serrano M. Kroemer G. (2013). The hallmarks of aging. *Cell* 153 1194–1217. 10.1016/j.cell.2013.05.039 23746838 PMC3836174

[B22] Marioni R. E. Shah S. McRae A. F. Chen B. H. Colicino E. Harris S. E. (2015). DNA methylation age of blood predicts all-cause mortality in later life. *Genome Biol.* 16:25 10.1186/s13059-015-0584-6 PMC4350614 25633388

[B23] Martin P. Jazwinski S. M. Davey A. Green R. C. Macdonald M. Margrett J. A. (2014). APOE 4, rated life experiences, and affect among centenarians. *Aging Ment. Health* 18 240–247. 10.1080/13607863.2013.827624 23998924 PMC3946882

[B24] Matteini A. M. Fallin M. D. Kammerer C. M. Schupf N. Yashin A. I. Christensen K. (2010). Heritability estimates of endophenotypes of long and health life: the Long Life Family Study. *J. Gerontol. A Biol. Sci. Med. Sci.* 65 1375–1379. 10.1093/gerona/glq154 20813793 PMC2990267

[B25] McArdle J. J. (2011). Longitudinal dynamic analyses of cognition in the health and retirement study panel. *Adv. Stat. Anal.* 95 453–480. 10.1007/s10182-011-0168-z 25598848 PMC4293541

[B26] Milot E. Morissette-Thomas V. Li Q. Fried L. P. Ferrucci L. Cohen A. A. (2014). Trajectories of physiological dysregulation predicts mortality and health outcomes in a consistent manner across three populations. *Mech. Ageing Dev.* 14 56–63. 10.1016/j.mad.2014.10.001 PMC4310774 25454986

[B27] Mitnitski A. B. Mogilner A. J. Rockwood K. (2001). Accumulation of deficits as a proxy measure of aging. *Sci. World J.* 1 323–336. 10.1100/tsw.2001.58 PMC6084020 12806071

[B28] Papenberg G. Lindenberger U. Backman L. (2015). Aging-related magnification of genetic effects on cognitive and brain integrity. *Trends Cogn. Sci.* 19 506–514. 10.1016/j.tics.2015.06.008 26187033

[B29] Park D. C. Bischof G. N. (2013). The aging mind: neuroplasticity in response to cognitive training. *Dialogues Clin. Neurosci.* 15 109–119. 23576894 10.31887/DCNS.2013.15.1/dpark PMC3622463

[B30] Perna L. Zhang Y. Mons U. Holleczek B. Saum K. U. Brenner H. (2016). Epigenetic age acceleration predicts cancer, cardiovascular, and all-cause mortality in a German case cohort. *Clin. Epigenetics* 8:64 10.1186/s13148-016-0228-z PMC4891876 27274774

[B31] Rockwood K. Andrew M. Mitnitski A. (2007). A comparison of two approaches to measuring frailty in elderly people. *J. Gerontol. A Biol. Sci. Med. Sci.* 62 738–743. 17634321 10.1093/gerona/62.7.738

[B32] Rockwood K. Mitnitski A. (2007). Frailty in relation to the accumulation of deficits. *J. Gerontol. A Biol. Sci. Med. Sci.* 62 722–727. 17634318 10.1093/gerona/62.7.722

[B33] Rowe J. W. Kahn R. L. (1997). Successful aging. *Gerontologist* 37 433–440. 9279031 10.1093/geront/37.4.433

[B34] Sebastiani P. Thyagarajan B. Sun F. Schupf N. Newman A. B. Montano M. (2017). Biomarker signatures of aging. *Aging Cell* 16 329–338. 10.1111/acel.12557 28058805 PMC5334528

[B35] Seeman T. E. McEwen B. S. Rowe J. W. Singer B. H. (2001). Allostatic load as a marker of cumulative biological risk: MacArthur studies of successful aging. *Proc. Natl. Acad. Sci. U.S.A.* 98 4770–4775. 10.1073/pnas.081072698 11287659 PMC31909

[B36] Shock N. W. (1967). Physical activity and the “rate of ageing.” *Can. Med. Assoc. J.* 96 836–842. 6020886 PMC1936152

[B37] Sierra F. (2016). The emergence of geroscience as an interdisciplinary approach to the enhancement of health span and life span. *Cold Spring. Harb. Perspect. Med.* 6 a025163. 10.1101/cshperspect.a025163 PMC4817738 26931460

[B38] Sleimen-Malkoun R. Temprado J. J. Hong S. L. (2014). Aging induced loss of complexity and dedifferentiation: consequences for coordination dynamics within and between brain, muscular and behavioral levels. *Front. Aging. Neurosci.* 6:140 10.3389/fnagi.2014.00140 PMC4073624 25018731

[B39] Vozza A. Parisi G. De Leonardis F. Lasorsa F. M. Castegna A. Amorese D. (2014). UCP2 transports C4 metabolites out of mitochondria, regulating glucose and glutamine oxidation. *Proc. Natl. Acad. Sci. U.S.A.* 111 960–965. 10.1073/pnas.1317400111 24395786 PMC3903233

