# Supplementary material for: Metabolic and Genetic Markers of Biological Age
Source: Front Genet. 2017 May 23;8:64. doi: 10.3389/fgene.2017.00064 (PMC5440459; doi:10.3389/fgene.2017.00064)
Supplement: Supplementary file 1 [file Table_1.PDF]

Supplementary Table 1. List of 34 variables used to construct the frailty index FI_{34}.

No.	Name	Description	Numeric code
1	adrdz	You've been told that you have an adrenal disease	0, 1
2	anemia	You've been told that you have anemia	0, 1
3	angina	You've been told that you have angina	0, 1
4.	asthma	You've been told that you have asthma	0, 1
5	balance	Standing for 10 sec. with one foot behind the other	0, 1[a]
6	bathing	You need assistance when bathing	0, 1
7	bmi	Body mass index (BMI)	0, 0.5, 1[b]
8	bronch	You've been told that you have bronchitis	0, 1
9	cataracts	You've been told that you have cataracts	0, 1
10	chair	Number of stand-ups from chair without using arms	0, 1[c]
11	conghrtf	You've had congestive heart failure	0, 1
12	copd	You've been told that you have COPD	0, 1
13	diabetes	You've been told that you have diabetes	0, 1
14	dressing	You need assistance when dressing	0, 1
15	emphy	You've been told that you have emphysema	0, 1
16	feeding	You need assistance when eating	0, 1
17	fhoca	A first-degree relative has had cancer	0, 1
18	gds	Geriatric depression scale (GDS)[[72, 73]	0, 0,5, 1[d]
19	hattack	You've had a heart attack	0, 1
20	hbp	High blood pressure (based on SBP and DBP readings)	0, 0.33, 0.66, 1[e]
21	hchol	You've been told that you have high cholesterol	1.00
22	hhbp	You have had high blood pressure before	0, 1
23	hrtmur	You've been told that you have a heart murmur	0, 1
24	hrtprb	You've been told that you have a heart problem	0, 1
25	kidndz	You've been told that you have a kidney disease	0, 1
26	liverdz	You've been told that you have a liver disease	0, 1
27	mmse	Mini-mental state exam (MMSE)[74, 75]	0, 0.25, 0.5, 0.75, 1[f]
28	osteo	You've been told that you have osteoporosis	0, 1
29	seiz	You've had a seizure	0, 1
30	selfrated	Self-rating of health	0, 0.25, 0.5, 0.75, 1[g]
31	stroke	You've had a stroke	0, 1
32	thydz	You've been told that you have a thyroid disease	0, 1
33	tia	You've had a TIA	0, 1
34	urininf	You've been told that you have a urinary infection	0, 1

COPD/copd, chronic obstructive pulmonary disease; SBP, systolic blood pressure; DBP, diastolic blood pressure; tia/TIA, transient ischemic attack. All binary variables were coded numerically: '0' for the absence of the deficit and '1' for its presence except where noted otherwise: [a] 0 if balanced for 10 seconds, otherwise,1; [b] 0 if $18.5 \le x < 25$, where x=weight (kg)/(height in meters)^2, 0.5 if $25 \le x < 30$, otherwise, 1; [c] 0 if one can stand up from chair at least once, otherwise 1; [d] 0 if $0 < x \le 5$, where x is the final score of the test, 0.5 if $6 < x \le 10$, 1 if $x > 10$; [e] 0 if $x < 80$ and $y < 120$, where x=diastolic pressure and y=systolic pressure, 0.33 if $80 \le x \le 89$ or $120 \le y \le 139$, 0.66 if $90 \le x \le 99$ or $140 \le y \le 159$, 1 if $x \ge 100$ or $y \ge 160$. This coding is based on the categories of blood pressure levels according to the National Heart Lung and Blood Institute; [f] 0 if $24 \le x$, where x is the final score of the test, 0.25 if $20 < x < 24$, 0.5 if $18 \le x \le 20$, 0.75 if $10 \le x \le 17$, and 1 if $x < 10$; [g] 0 = Excellent, 0.25 = Very good, 0.5 = Good, 0.75 = Fair, 1 = Poor. Reproduced with permission from Kim, S., Welsh, D.A., Cherry, K.E., Myers, L., and Jazwinski, S.M. (2013). Association of healthy aging with parental longevity. *Age (Dordr)* 35(5), 1975-1982. doi: 10.1007/s11357-012-9472-0 and Kim, S., and Jazwinski, S.M. (2015). Quantitative measures of healthy aging and biological age. *Healthy Aging Res* 4. doi: 10.12715/har.2015.4.26.
